# A marker-free genome editing method in *S. pombe* using the *delitto perfetto* approach.

**DOI:** 10.17912/micropub.biology.000997

**Published:** 2023-10-09

**Authors:** Karine Fréon, Sarah A.E. Lambert, Kirill S. Lobachev, Anissia Ait Saada

**Affiliations:** 1 Institut Curie, PSL Research University, UMR3348, 91400 Orsay, France.; 2 Paris-Saclay University, 91400 Orsay, France.; 3 CNRS, UMR3348, 91400 Orsay France. Equipe Labellisée ligue contre le Cancer.; 4 School of Biological Sciences, Georgia Institute of Technology, Atlanta, Georgia, United States

## Abstract

The fission yeast, like budding yeast, offer an easy manipulation of their genome, despite their distinct biology. Most tools available in budding yeast are also available in fission yeast in versions taking into account the features of each organism. The *delitto perfetto* is a powerful approach, initially developed in *S. cerevisiae* , for *in vivo* site-directed mutagenesis. Here, we present an adaptation of the approach to *S. pombe* manipulation and demonstrate its applicability for a rapid, marker-free and efficient *in vivo* site-directed mutagenesis and N-terminal tagging of nonessential genes in fission yeast.

**Figure 1. Application of the delitto perfetto approach to S. pombe .**
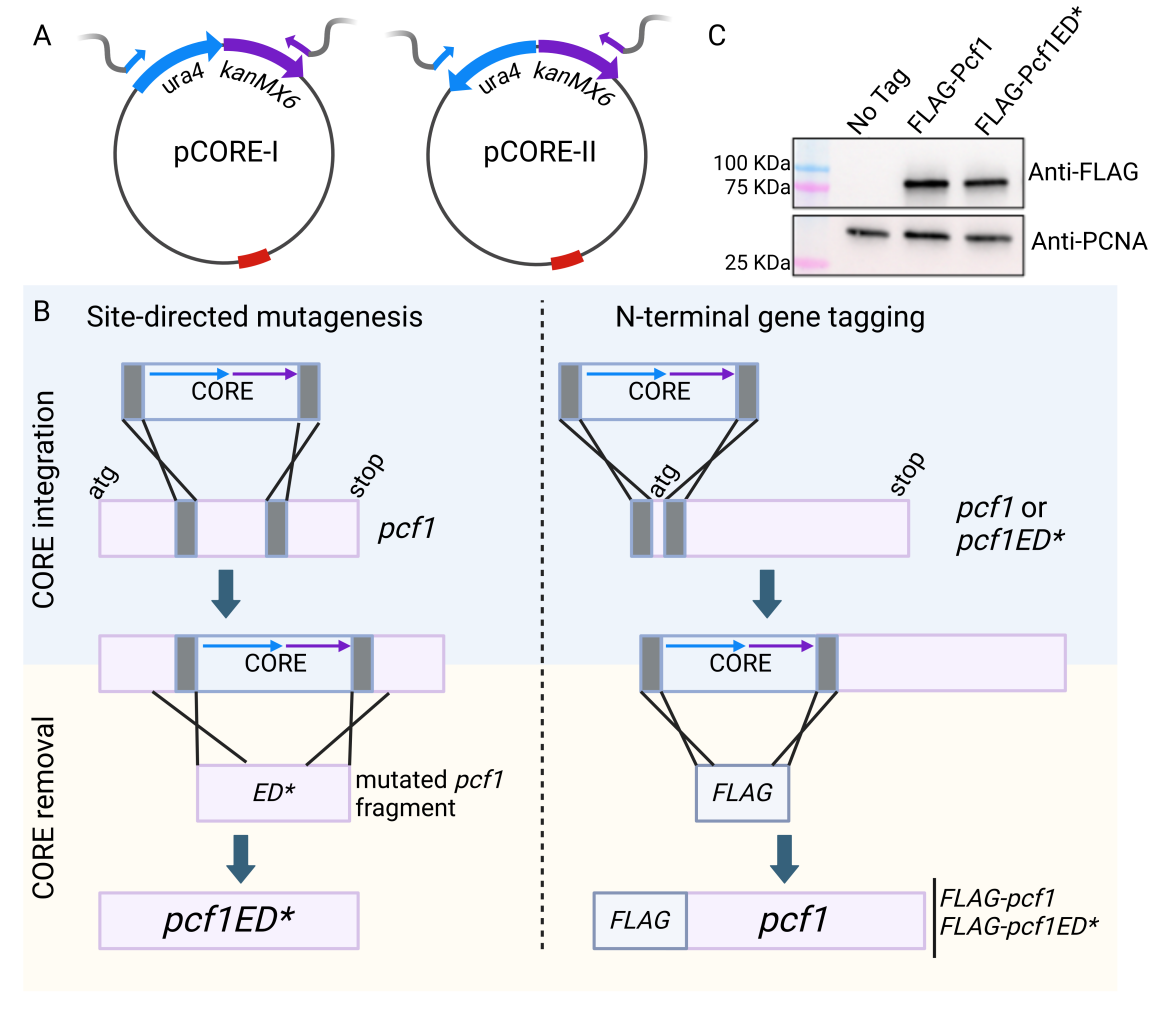
**A.** CORE cassette design. The *ura4* gene was cloned into the *kanMX6* -containing plasmid (pFA6a-kanMX6) following two orientations. In pCORE-I, the *ura4* and *kanMX6* genes are expressed co-directionally whereas in pCORE-II the two genes are divergently expressed. The primers used for the amplification of the CORE cassette are represented as a blue and purple arrows annealed to the *ura4* and *kanMX6* genes, respectively. The gray tails correspond to a 100 bp sequence to target the genomic region of interest. **B.** Strategy for using the *delitto perfetto* approach for site-directed mutagenesis (left panel) and N-terminal gene tagging (right panel). *pcf1* mutagenesis and N-terminal tagging are given as an example (see text for more details). The grey boxes correspond to the gene-specific sequences bordering the insertion site of the CORE cassette (grey tails in A). **C.** Western blot analysis of FLAG-Pcf1. This figure was created with BioRender.com.

## Description

The function of specific genes can be deduced by targeted modifications through gene manipulation. These include, for example, gene knockouts, mutations or overexpression. The *delitto perfetto* approach is a two-step process used for targeted mutagenesis with oligonucleotides, initially developed in *S. cerevisiae* (Storici et al., 2001) . As indicated by its name, it allows to modify any chromosome region without leaving a trace of foreign DNA, as opposed to other systems such as the Cre/lox system where the lox sequences are bound to be part of the genome (Watson et al., 2008) . It relies on the integration of a COunterselectable REporter (CORE) cassette (containing positive and negative selectable markers) and its replacement with the appropriate synthetic oligonucleotides or DNA fragments using the yeast’s homologous recombination (HR) machinery (Storici and Resnick, 2006) . The *delitto perfetto* approach offers a series of applications for site-directed mutagenesis and chromosome modifications (Stuckey and Storici, 2013) . The practicality and adaptability of *delitto perfetto* make it the technique of choice for efficient and precise manipulation of genes in *S. cerevisiae* .

*S. pombe* lends itself to genetic manipulation and gene targeting methods using modular, PCR-based approaches are available. The use of a double-marker system is known to facilitate the construction of unmarked alleles in fission yeast (Mudge et al., 2012) . Here, we adapted the *delitto perfetto* approach for genetic manipulation of the *S. pombe* genome. As a proof of concept, we show the application of the *delitto perfetto* for the generation of Pcf1 mutated forms. Pcf1 is one of the 3 subunits of the Chromatin Assembly Factor 1 (CAF-1) complex, a histone H3-H4 chaperone involved in nucleosome assembly coupled to DNA replication (Dohke et al., 2008; Pietrobon et al., 2014) . The *pcf1* mutations were designed to answer questions addressed in (Ouasti et al., 2023) . Each of the Pcf1 mutants harbors multiple mutations among which the Y340A-W348A mutant (called Pcf1ED* and shown as an example here). The strength of the *delitto perfetto* approach is that it allows generating any complex mutant in only two rounds of transformation without the need to resort to cloning. Indeed, another marker-free allele replacement approach (pop-in, pop-out allele replacement) has been developed in *S. pombe* but requires cloning of the allele of interest (Gao et al., 2014) .

Since the method allows for direct selection of any chromosomal modification, we applied *delitto perfetto* for N-terminal gene tagging. The choice between N-terminal and C-terminal tagging depends on how the functionality and/or expression of the resulting protein is affected. N-terminal tagging can however be challenging since it is not possible to use a selectable maker to follow the integration of the tag without disturbing the gene expression. We show here the application of *delitto perfetto* for efficient and rapid N-terminal tagging of nonessential genes in *S. pombe* , with Pcf1 serving as an illustrative example.

**Generation of the *S. pombe* CORE cassette:** The CORE cassette is composed of the *S. pombe* counter-selectable marker *ura4* gene and the reporter gene *kanMX6* (geneticin or G418 resistance gene). The selectable marker *ura4* is widely used for PCR-based gene replacement experiments (Grimm et al., 1988) since prototrophic strains and auxotrophic mutants can be selected on media lacking uracil and 5-FOA-containing media, respectively. The *ura4* gene (corresponding to the Bst1107I fragment) has been cloned into the pFA6a-kanMX6 plasmid (addgene #39296) at the SmaI restriction site. Because of a blunt-end to blunt-end ligation, *ura4* was inserted in two orientations relative to *kanMX6* , resulting in pCORE-I and pCORE-II plasmids ( Figure 1A ). Either plasmid can be used as a template for the amplification of the CORE cassette flanked with 100 bp homology corresponding to the region of interest, using the following primer design:

Forward 5’ (gene-specific region, ~100bp) GAATTCGAGCTCGTTTAAAC 3’

Reverse 5’ (gene-specific region, ~100bp) CAGGTCGACGGATCCCCTAC 3’

**Integration of the CORE cassette:** The amplified CORE fragment is used for yeast transformation and its integration at the desired location is driven by the 100 bp flanking homology ( Figure 1B ). The integration of the CORE cassette was selected on media containing G418. G418 ^R^ URA ^+^ clones were then tested for the correct cassette integration *via* classic colony PCR. For *pcf1* targeting for mutagenesis, the efficiency of the CORE cassette integration at the *pcf1* locus corresponded to 83% (20 out of 24 tested clones). Since the *pcf1* mutants should contain several mutations distributed in a region spanning up to 60 nucleotides, the CORE cassette was inserted in the *pcf1* gene in a way that disrupts a sequence of ~ 70-120 bp (corresponding to the region to mutate). For the N-terminal tagging, the CORE cassette was inserted right between the end of the 5’UTR and the second *pcf1* codon, thus deleting the ATG start codon. Once a CORE cassette is inserted at a given locus, different mutations can be generated at that locus or its vicinity.

**CORE cassette removal:** During this step, the CORE cassette is swapped with the fragment of interest to create the desired sequence ( Figure 1B ). For single point mutations or complete deletion of a gene, synthetic oligonucleotides can be used (Storici et al., 2001) . Here, since the fragment to use for CORE replacement should contain the 70-120 bp plus at least 100 bp homology from both sides, we preferred to synthesize ~ 500 bp fragments. This has the advantage of increasing the length of the homology region to enhance gene targeting efficiency (Krawchuk and Wahls, 1999) . The CORE cassette-containing strain was transformed with these fragments and the cassette replacement was monitored by selecting 5-FOA resistant cells. The resulting strains were tested for their sensitivity to G418 to eliminate the ones experiencing a spontaneous mutation in the *ura4* gene since the probability of spontaneous mutations in both *ura4* and *kanMX6* is extremely low. For the given example, 28 5-FOA-resistant clones were obtained, out of which 9 (32%) were also G418 sensitive. The correct replacement of the CORE cassette was assessed by PCR and 100% of the 5-FOA ^R^ G418 ^S^ strains were devoid of the CORE cassette. The mutations were verified by sequencing.

For N-terminal tagging, the CORE cassette-containing strains were transformed with a FLAG sequence flanked with a ~ 100 bp homology corresponding to the insertion region, *i.e.* 100 bp upstream of the *pcf1* open reading frame on 5’ and the 100 bp just downstream the ATG start codon on 3’ ( Figure 1B ). A 15 bp linker sequence (5G, encoding five glycines) was included between the FLAG sequence and *pcf1* (see Methods section for the primer design). The FLAG fragment was amplified from the pFA6a-G9-5FLAG-hphMX6 plasmid (addgene 49185). Successful cassette removal was verified as described and protein expression was assessed by Western blot analysis ( Figure 1C ).

**Conclusion:** The *delitto perfetto* approach is widely used in budding yeast and offers a wide variety of genome modifications ranging from point mutations to complex rearrangements (Stuckey and Storici, 2013) . We adapted the approach and show that the *delitto perfetto* is an easy and efficient approach for *in vivo* mutagenesis in fission yeast. The adaptability of the DNA fragments to create any mutation in nonessential genes and the use of a double reporter system significantly reduces the labor to create complex mutations or generate N-terminal tagged genes. We also used the approach for gene knockouts (Ait Saada et al., 2022) . It becomes particularly useful to resort to the *delitto perfetto* for i) creating strains with several gene deletions without exhausting the pool of available selectable markers and ii) tagging nonessential genes refractory to C-terminal tagging. One caveat is that several rounds of transformations, known to be a mutagenic process, are required to generate the strains. To circumvent this, working with several clones or backcrossing the transformed strains should be considered. Since the CORE cassette integration and removal rely on the HR machinery of the cells, the starting strains should be HR proficient and *ura4* deleted.

## Methods

*Yeast strains and media*

The yeast strain used for the *delitto perfetto* approach harbors the following genotype: *h- ura4-D18 leu1-32 ade6-704* . Cells were grown at 30°C in YES or in supplemented Edinburgh minimal medium (EMM) without uracil as previously described (Moreno et al., 1991) .

*Primers used for site-directed mutagenesis and N-terminal tagging of pcf1*

**Table:** 

**Name**	**Sequence**	**To amplify**
KF194	*AAGAGAACAGGAAAAAATCGCCGCTAAGAAAATGAAAGAATTAGAGAAATTAGAGAAGGAGCGGATTCGATTGCAGGAACAACAAAGGCGGAAGGAGGAG* **GAATTCGAGCTCGTTTAAAC**	CORE cassette for KER mutation from pCORE-I plasmid
KF195	*TCAGTTCATCCGTTTTGTCAGCTACAAAATTTTCGTTTGGCGCGATTCGTTTCTCAACTCCTTTGGTGAAAAAGTTGTTTAACTTCAATTGTTGTCTTTC* **CAGGTCGACGGATCCCCTAC**	
KF198	*TCAGGATGTTCGCCCTCCATATTTTGGATCTTACACGAAAACCCATTCCCATGGTTCTAACGTTTTATTGAATCCCTGGCTTGAAGATGAAGACATAGAC* **GAATTCGAGCTCGTTTAAAC**	CORE cassette for ED mutation from pCORE-I plasmid
KF199	*TTGAATCCCAAACCGGACCTTCTACGATTACCTCTAATGGGCCGGAGGAACGATGGGTATTAGAAGCATTGACACTATCCTTGTCATCATCCTCATCATC* **CAGGTCGACGGATCCCCTAC**	
Nter-coreF	*CTTTATCCTCTTTATTCGATCAGAGCGTCCGTTAAAACAACTTACGCACGGTTTTTAGCCTCCCTTAGGCTTGTTATTGATATTCGCAGCATTGTCTATG* **GAATTCGAGCTCGTTTAAAC**	CORE cassette for N-terminal tagging pCORE-I plasmid
Nter-coreR	*AACTCACTGATAAAGACGTAATGTCAGTGGAAGAGCTACATAACTCATTTCCCTTATTACTAGTAGAGGCCGCGACATCTGAATCAACACTTTCACTATT* **CAGGTCGACGGATCCCCTAC**	
FLAG-pcf1F	*TTTATCCTCTTTATTCGATCAGAGCGTCCGTTAAAACAACTTACGCACGGTTTTTAGCCTCCCTTAGGCTTGTTATTGATATTCGCAGCATTGTCT* **GGTGGAGGTTTAATTAATCATATG**	The FLAG sequence from pFA6a-G9-5FLAG-hphMX6 plasmid
FLAG-pcf1R	*AGACGTAATGTCAGTGGAAGAGCTACATAACTCATTTCCCTTATTACTAGTAGAGGCCGCGACATCTGAATCAACACTTTCACTATT* TCCACCTCCACCTCC **AAGTGGCGCGCCCATATG**	

*Italic* : sequence homologous to *pcf1* . **Bold** : plasmid-specific sequence. Underlined : G5 linker sequence.

*Yeast transformation*

2x10 ^8^ cells from exponentially growing culture in YES were collected and washed twice in water and once in 0.1M LiOAc. Cells were pelleted and resuspended in 100 µl of 0.1M LiOAc and 2 µl of heat-denatured herring sperm DNA (D7290, Sigma Aldrich) and ~ 1 µg of purified PCR product (corresponding to the CORE cassette or the FLAG fragment) were added. After 10 min of incubation at room temperature, 260 µl of 40% PEG (PEG4000) was added. The cell suspension was then incubated for 1h at 30°C. 43 µl of DMSO were then added and heat shock was performed at 42°C for 5 min. Cells were collected by centrifugation at low speed (2500 rpm) and washed once. Pellets were resuspended in fresh YES, plated on YES plates and the plates were incubated overnight at 30°C. The plates were then replica-plated on the appropriate selective medium (YES+G418 for the CORE cassette integration or YES+5FOA for its removal).

*Protein extraction and western blot analysis*

1x10 ^8^ cells from exponentially growing culture in YES were collected for protein extraction. Cell pellets were washed twice in 1 ml of stop buffer (50 mM NaF, 10 mM NaN3 in PBS 1X), washed once in 1 ml of 20% trichloroacetic acid (TCA) and then resuspended in 200 μl of 20% TCA. Glass beads (G8772, Sigma Aldrich) were added for cell wall disruption with the Precellys bead beating homogenizer in the cold room with two rounds of the following program: 4 cycles {20 sec ON 10 000 rpm, 120 sec OFF}, and a 10 min wait in between. After adding 400 μl of 5% TCA, the cell lysate was recovered without the beads spun at high speed for 5 min. The pellets were resuspended in 200 μl of TCA buffer (1x SDS loading buffer, 0.2M Tris-HCl pH 8). The samples were boiled at 95°C for 5 min a spun briefly prior to western blot analysis. The proteins were separated on a 4-15% MP TGX Stain-Free gel (4568085, BioRad) and transferred to a nitrocellulose membrane using a transblot turbo system (BioRad). The FLAG-Pcf1 protein was probed with an anti-FLAG antibody (1:1000, F1804 Sigma Aldrich). PCNA (revealed by the 1:500 anti-PCNA antibody, SC-53 Santa Cruz) was used as a loading control.
